# Renal and Adrenal Tumours

**DOI:** 10.1038/sj.bjc.6701274

**Published:** 2003-10-14

**Authors:** A Protheroe

**Affiliations:** 1 Cancer Research UK, Oxford

‘To provide a single, comprehensive source of information on renal and adrenal tumours in a single volume that will be of practical interest for students, residents, physicians and researchers alike’ is one of the objectives of the editors of this book. As with all aspects of management of patients with cancer, there is a vast amount of literature and finding the definitive tome is difficult. This book I believe has found a niche and brings together a wealth of information on these tumours ranging from historical perspectives through to both surgical and medical management together with some experimental overviews and relatively recent research. Certainly, all the information is available in many other books but not in one comprehensive volume.

The authors themselves are eminent in their own fields, which gives a book such as this immediate credibility (although there are a few omissions). They are also fairly cosmopolitan and therefore views from both Europe and the United States are well represented. The book is divided into several sections covering anatomy, epidemiology and pathology through molecular genetics and immunobiology to imaging and management and finally renal cancer metastatic disease. Adrenal tumours are covered in one section in part 2 of the book.

I found it a very readable informative book covering a wide range of topics both surgical and nonsurgical, and providing a wealth of information which hitherto was difficult to find in one place. It therefore appeals to a wide audience. As a physician, I found the chapters on the history of surgery for renal cancer very enjoyable and interesting as were the overview chapters on surgical management. However, I did find some chapters on the medical management lacking some of the current controversies and were not quite as in depth as I would have liked. I would also have liked to see a more rounded view on some of the systemic treatment, and a true reflection on the current issues. However, that probably does not matter as it does represent a very good overview and I cannot be too critical within my own specific area. I was also disappointed to find a lack of detail in managing the rare metastatic adrenal tumours, particularly with regard to systemic treatments, although the diagnosis and surgical treatment were comprehensively covered.

As with most books written by multiple authors, some chapters are stronger than others, there are differences in styles of writing and it can be a bit repetitive in places. However, this is a reference book to dive in and out of and repetition can be helpful.

The objectives of the editors I think were achieved well and I would thoroughly recommend this book. In terms of the audience it always will be a niche market, but any healthcare professional with an interest in renal and adrenal tumours would do well to use this as a source of information. It should become the authoritative textbook for these tumours, particularly if subsequent editions keep abreast of this rapidly changing field.

